# Protecting the Offspring, the Gift of Maternal Immunization: Current Status and Future Perspectives

**DOI:** 10.3390/vaccines10111953

**Published:** 2022-11-18

**Authors:** Theano Lagousi, Despoina Gkentzi, Maria Geropeppa, Panagiota Tsagkli, Vana Spoulou

**Affiliations:** 1 Immunobiology and Vaccinology Research Laboratory and Infectious Diseases Department “MAKKA”, First Department of Paediatrics, “Aghia Sophia” Children’s Hospital, Athens Medical School, 11527 Athens, Greece; 2 Department of Pediatrics, University of Patras Medical School, 26504 Patras, Greece

**Keywords:** maternal immunization, influenza, pertussis, SARS-CoV-2, fetal immune system, neonates, infants, immune system priming

## Abstract

Pregnancy is characterized by immunological alterations in pregnant women that permit the growth of a semi-allogenic fetus, resulting in greater susceptibility of childbearing women to infections. Furthermore, due to the immaturity of the immune system of neonates, a protection gap is present in early life, leaving neonates and infants vulnerable to infectious diseases with increased morbidity and mortality. Maternal immunization against influenza, pertussis, and, in the context of the COVID-19 pandemic, SARS-CoV-2 has been implemented in several countries, with beneficial effects on both the mother and the offspring. The main protective mechanism of vaccination during pregnancy is transplacental transfer of maternal antibodies. However, recent evidence has implied that the fetal immune system may be influenced beyond passive immunity. This review sheds light on the current status of the routinely administered vaccinations during pregnancy, focusing on the impact of maternal immunization on the priming of the fetal immune system and suggesting future perspectives for the optimization of vaccination strategies.

## 1. Introduction

Pregnant women are more susceptible to common infections. This is due to attenuated immune responses to antigens, driven by the immunological changes occurring during gestation, in order to support pregnancy and tolerance of a semi-allogenic developing fetus [1]. Consequently, infections during pregnancy often result in severe maternal disease, increased maternal mortality, and associated pregnancy complications, i.e., spontaneous abortion and pre-term birth [2,3]. Neonates are also vulnerable to certain infections due to their naive immune system [4]. Furthermore, vaccination of full-term and preterm neonates is challenging due to their immature immune system and inefficient immune response to vaccine antigens [5].

Vaccination during pregnancy is a well-established strategy to protect both the mother and the developing fetus from the corresponding infections. Currently, vaccines routinely administered during pregnancy include the inactivated influenza vaccine and the combined tetanus toxoid, reduced diphtheria toxoid, and acellular pertussis vaccine (Tdap). The World Health Organization (WHO), the Advisory Committee on Immunization Practices (ACIP), and the American College of Obstetricians and Gynecologists (ACOG) recommend immunization with inactivated influenza vaccine for all pregnant women regardless of the stage of pregnancy, as well as for women of childbearing age [6,7]. In 2011, the ACIP recommended Tdap vaccination in the last trimester of pregnancy (27–36 weeks of gestation) for women that had never been vaccinated with Tdap before pregnancy [8]. This recommendation was extended in 2012 to all women of childbearing age, regardless of previous vaccination status, and has been implemented ever since [9,10]. Following the pertussis epidemic in 2012 in the UK, a temporary maternal vaccination program was launched in 2012 and is still ongoing due to continuing high numbers of pertussis cases [11]. In addition to the US and UK, maternal Tdap vaccination is currently recommended and government-funded in many countries globally [12,13,14,15]. Remarkably, in the setting of COVID-19 pandemic during the last 2 years, pregnant women are currently strongly advised to get vaccinated against SARS-CoV-2, to protect both themselves and their infants [16]. Except for the routinely administered vaccines, several vaccines can be safely used in pregnancy under specific circumstances. In the context of an epidemic, before traveling or after exposure, pregnant women can be vaccinated against hepatitis B, Neisseria meningitidis with the meningococcal polysaccharide vaccines, and polio with the inactivated virus vaccine (IPV) [17].

Beneficiary effects of maternal immunization for both mothers and fetuses have been thoroughly described. Maternal immunization confers protection to both mothers and fetuses by augmenting concentrations of maternal antibodies [18]. Thus, the quantity of antibodies transferred through the placenta to the fetus is enhanced, leading to more effective protection of the neonate during the period of the highest vulnerability until routine infant vaccinations are initiated [19]. Several factors may affect this process. Firstly, the timing and quality of the IgG transplacental transfer are of paramount importance for the development of maternal immunization strategies. While maternal IgG antibodies are transplacentally transferred throughout pregnancy, the majority of the transfer occurs in the third trimester of gestation. This is possibly due to an increase in the surface area of IgG uptake in placenta with higher gestational age [18]. It has been established that the transplacental transfer of maternally derived antibodies already begins from the first trimester of gestation and increases longitudinally, where the maximum IgG transfer occurs after the 28th gestational week [20]. In addition, maternally derived IgA and IgG are also transferred after birth in the colostrum and the breastmilk, enhancing the immunological response of the offspring in the first months of life [21,22].

**Figure 1:**
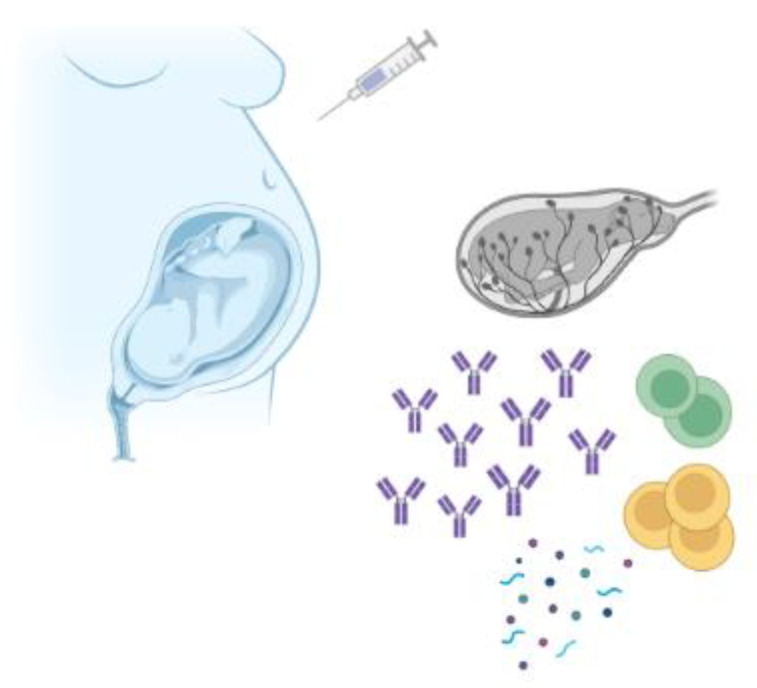
Maternal vaccination may influence the fetal immune system beyond passive transplacental transfer of vaccine-specific antibodies. Evidence suggests that vaccination during pregnancy could prime fetal B cells and T cells. The role of cytokines is currently under investigation. Created with BioRender.com.

Importantly, it has been lately reported that the fetal and neonatal immune system may be affected beyond the passive immunity induced through IgG transfer; transfer of immune cells from the mother to the fetus has been proposed, as shown in Figure 1 [23,24]. It is possible that in utero priming following maternal immunization may provide additional, antibody-independent protection to the neonate. Current evidence suggests that fetal and neonatal immune system may be primed in response to maternal vaccinations, with confounding effect on subsequent neonate and infant B-cell repertoire [25]. Maternal cells have been identified in fetal tissues already from the second trimester of gestation. These cells are transferred via the placenta and promote the generation of fetal T regulatory cells against certain maternal antigens, somehow priming the fetal immune system [24]. In support of this, fetal memory T cells against human immunodeficiency virus (HIV), hepatitis B, and hepatitis C have been detected in uninfected children of mothers with wildtype infection. The aforementioned findings suggest in utero exposure to maternally derived infectious antigens, during maternal wildtype infection, implying that a similar mechanism may occur following maternal vaccination [26].

Nonetheless, several issues remain to be elucidated. Although the aim of maternal vaccination is the increase in maternal antigen-specific antibody titers and antibodies transferred to the infant through the placenta, maternal antibodies could interfere with offspring immune response to subsequent routine immunizations. This so-called “immunological blunting effect” results in attenuated humoral responses in early or late infancy [27]. However, the clinical significance of blunting remains unclear. Furthermore, vaccines during pregnancy may have “nonspecific effects”, by inducing modifications in the fetal immune system in an independent way to antigen-specific adaptive responses [28].

In this review, we aim to summarize current data on routinely administered immunizations during pregnancy and their effect on early life immune responses. In-depth understanding is crucial to achieve better infant survival rates and further optimize maternal and infant vaccination strategies.

## 2. Maternal Immunization against Influenza

Influenza infections are more severe during pregnancy due to changes in cardiorespiratory function of pregnant women, leading to high rates of morbidity and mortality among this vulnerable population, which progressively increase during pregnancy [29]. Notably, mortality rates surged among pregnant women (up to 45%) amidst major global influenza outbreaks [30]. Infection-related adverse events also occur among fetuses and neonates, with increased risk of miscarriage, preterm birth, low-birthweight neonates, and neonatal death [31]. Furthermore, both inactivated and live influenza vaccines are not currently indicated for children below 6 months due to safety reasons and impaired immunogenicity [32]. Thus, young infants are left unprotected during a period when they are more susceptible to severe complications. Currently, the absence of influenza epidemic during COVID-19 pandemics is expected to negatively affect infants in the following influenza seasons. Mothers most likely would not be capable of transferring antibodies to their offspring, as they have neither been exposed to strains of the virus, nor been routinely immunized. Hence, the launch of strategies that promote maternal vaccination against influenza worldwide is of paramount importance.

### 2.1. Maternal Immunization Effectiveness and Immunogenicity in Animals and Humans

Maternal immunization is critical to protect embryos in utero or newborns postnatally from adverse effects of influenza infection [33]. Immunization of gestating mice and ferrets with an inactivated vaccine containing H3N2, H2N2, H1N1, or H5N1 antigens provided enhanced passive protection to neonate animals [34,35]. Prospective studies in humans with laboratory-confirmed influenza showed that maternal vaccination not only reduces risk of influenza infection in infants (up to 63%), but also protects infected infants from severe disease [36].

Taking into consideration the frequent genetic modifications of the influenza virus, the WHO, the ACIP, and the ACOG recommended annual immunization with a quadrivalent inactivated influenza vaccine for all pregnant women regardless of pregnancy trimester, as well as for women of childbearing age or in the postpartum phase [37,38,39]. Nevertheless, only 61.2% of pregnant women in the US were vaccinated in 2020, and this percentage ranged globally from 1.7% to 95% [17]. Pregnancy is a state of relative immune suppression; thus, immunization during pregnancy has been estimated at 41–91% effectiveness in protecting newborns against influenza [40]. These results may be overestimated considering the protective impact of breastfeeding, as well as reduced risk of exposure to the influenza virus achieved by cocooning practices. Importantly, although a single dose of H1N1pdm09 vaccine was proven to induce seroprotection in most subjects, vaccines against pandemic viruses such as H5N1 display low immunogenicity. Thus, multiple immunizations with high antigen doses or addition of adjuvants are required to achieve seroprotective levels [41]. The development of influenza vaccines that are not affected by pregnancy-associated immune suppression is an urgent priority.

### 2.2. When to Vaccinate Mothers

Pregnant women are encouraged to be vaccinated against influenza during any trimester. However, flu vaccine administered to pregnant women in the late second or third trimester (after 20 weeks gestation) may offer two significant benefits. Firstly, the current subunit or split influenza vaccines induce a short-lived humoral immunity, which wanes at 6–7 months post vaccination, whereby a late vaccination would efficiently protect the mother until labor. Secondly, since infants are not vaccinated against influenza before 6 months of age, it is crucial to provide them with a robust passive immunity via transplacental transfer of maternal antibodies in utero or by breastmilk during infancy. Consistent with this, a meta-analysis documented that women vaccinated earlier in pregnancy had a greater increase in antibody titers compared to those vaccinated later [42]. Μοreover,, the first and early second trimesters of pregnancy, including implantation and placentation, are characterized by a proinflammatory environment, with potentially increased risk of adverse events to infectious and vaccine antigens [43,44,45]. Later, during the second trimester, the maternal immune system is predominated by T regulatory (Treg) cells in order to tolerate the semi-allogenic developing fetus [46]. Treg cells regulate exuberant immune responses and may inhibit the protective immunity induced by infection and vaccination [47]. During the late second and third trimester, the period of rapid fetal growth and development, hormonal changes, and exposure to fetal antigens, maternal immunity moves toward a more anti-inflammatory setting; thus, a more effective response to vaccine antigens is expected [1,48]. Nevertheless, infants of mothers who were vaccinated just 2 weeks before labor were not seroprotected against influenza [33,49]. This finding implies that the influenza vaccine should be administered to pregnant women at least 15 days before labor to achieve maximum neonatal protection. In a study conducted in Mali during three influenza seasons, the overall vaccine effectiveness (VE) of trivalent vaccine for infants against influenza was 70.2% in the first 4 months of age but reduced to 37.3% in the fifth and sixth months of age [50]. Similarly, in a trial from South Africa in 2011–2012, the VE was 85.6% in infants aged below 8 weeks, 25.5% in infants aged 8–16 weeks, and 30.3% in infants 16–24 weeks of age [51]. Hence, further research is required to define the optimal timing to achieve the highest maternal vaccine-derived IgG antibodies.

### 2.3. Passive Immunity and in Utero Priming

The primary mechanism of transferred passive immunity during gestation is an interplay between the Fc part of IgG antibodies and the neonatal Fc-receptor (FcRn), expressed on placental syncytiotrophoblasts. FcRn is prone to binding more favorably to IgG1 compared to other subclasses, mainly during the last 4 weeks of the pregnancy, by better promoting natural killer function [52]. Notably, antigen-specific antibodies against influenza are mainly ΙgG1, effectively protecting the infant in the first months of life. Remarkably, Pou et al. showed that the concentration of IgG specific antibodies was not correlated with factors such as gestational age, maternal IgG concentration, and birthweight [53]. Thus, the role of other important determinants for IgG transfer, such as isotype differences, glycosylation patterns, or binding capacity to the FcRn, remains to be studied, to further optimize maternal and infant immunization strategies.

Although transplacental transfer of IgG during pregnancy is crucial for the protection of the newborn against influenza infection, evidence suggests that the fetal immune system may be influenced by maternal vaccination in more ways than the passive immunity provided through IgG transfer. As with infectious disease antigens [25], the fetal immune system may also be primed in utero to vaccine antigens to which the mother was vaccinated during pregnancy; however, research in this area is still limited. In a case series, influenza-specific IgM antibodies were detected in the cord blood of one out of eight infants born to immunized mothers [54]. Given that IgM cannot cross the placenta, such findings imply that fetal B lymphocyte sensitization may have occurred. More recently, Rastogi et al. reported that the influenza-specific cord T cells were mainly CD45RO^+^ [55]. However, it has been previously demonstrated that cord-blood T cells are usually predominantly naïve, due to their low expression of CD45RO [56,57]. Therefore, Rastogi et al.’s findings may suggest activation of the adaptive immunity in the fetus following maternal immunization against influenza, considering that these compartments of immune response present features of immunological effector memory. Nevertheless, it remains unclear whether fetal priming, to the extent that it occurs, may shape the subsequent postnatal vaccine or clinical response.

### 2.4. Blunting Effect

Despite the benefits of maternal antibodies transferred to the fetus, a blunting effect has been described [26]. Mechanisms of interference between maternal antibodies and the infant’s antibody titers in response to active vaccination and the duration of this effect remain unclear. It has been suggested that B-cell responses are mainly inhibited by the following: (i) antigen neutralization (live replicating vaccines) [58]; (ii) epitope masking inhibiting antigen binding and, thus, infant B-cell priming (as in antibody feedback regulation studies) [22,59]; (iii) active inhibition of infant B-cell activation by Fcg receptor IIB (FcgRIIB)-mediated signaling [18,60]; (iv) clearance of complexes of maternally derived antibodies and vaccine antigens through Fc-dependent phagocytosis [58,61]. It is widely considered that immunization in the presence of maternal antibodies essentially leaves CD4^+^ T cells unaffected but prevents B-cell activation and, thus, antibody responses [62].

Assessing the dampening or inhibition of antibody response following the flu vaccine in infants of immunized mothers is quite challenging, as no vaccine is recommended for infants <6 months of age; thus, studies on the safety and immunogenicity of influenza vaccines on this vulnerable population are limited. In general, the majority of infants <6 months with high maternal antibody titers who received influenza vaccine did not significantly increase antibody titers [63,64,65]. This antibody suppression was not validated by Groothuis et al., as all infants enrolled had low titers to the antigens tested before vaccination. Nonetheless, this study included many patients with bronchopulmonary dysplasia who were born prematurely and had limited transplacental transfer of maternal antibodies [66].

However, a recent study investigated long-term influence of maternal antibodies against influenza on infant response to influenza vaccination using several neonatal and infant immunization models. It was demonstrated that, in spite of intact CD4^+^ T effector cell responses, T follicular helper cells prematurely decline. Of note, the presence of high titers of maternal antibodies did not dampen B-cell activation, germinal center (GC) B-cell differentiation, and bona fide GC responses to neonatal immunization [67]. Therefore, successful activation and differentiation of neonatal and infant B cells into GC B cells do occur, even in the presence of very high titers of maternal antibodies. Maternally derived antibodies essentially control the B-cell repertoire leading the infant B cells to express distinct BCRs, binding to distinct epitopes of vaccine antigens, most likely non-immunodominant ones. Similarly, Zimmermann et al. concluded that there is no effect of maternal vaccination with a trivalent inactivated influenza vaccine on the antibody concentrations and the seroprotection rates at 7 and 13 months of age after the completion of the primary series and the 12 month vaccination [68].

## 3. Maternal Immunization against Pertussis

*Bordetella pertussis* is the causative pathogen of pertussis disease. Very young infants are at disproportional risk of severe complications, including secondary bacterial pneumonia, apnea, pneumothorax, dehydration, seizures, encephalitis, and death [69]. Therefore, maternal immunization is of great importance to protect the offspring from pertussis and its complications [70]. Pertussis-containing vaccines include whole-cell pertussis vaccines and acellular pertussis vaccines, which contain one or more pertussis antigens, including pertussis toxoid (PT), filamentous hemagglutinin (FHA), pertactin (PRN), and fimbrial proteins 2 (FIM2) and 3 (FIM3) [71]. Currently, acellular pertussis vaccines are recommended in most developed countries, since whole-cell pertussis-containing vaccines were associated with increased frequency of local and systemic adverse events. The use of whole-cell pertussis vaccines is limited to some low- and middle-income countries [72]. As the pertussis vaccine is not administered mono-component, but only combined with diphtheria and tetanus toxoids, vaccination in pregnancy is performed with the Tdap vaccine. Infants also receive the DTaP vaccine, containing the same antigens as Tdap but in different quantities [73].

### 3.1. Maternal Immunization Immunogenicity and Effectiveness in Animals and Humans

Maternal antibodies effectively protected their offspring from *B. pertussis* in animal studies [74,75]. The humoral response after single Tdap immunization among pregnant women is evanescent and may not confer protection in subsequent pregnancies [76,77,78,79]; therefore, according to the WHO and the ACIP, the Tdap vaccine should be administered to all pregnant women in every pregnancy, regardless of the previous vaccination status [10]. Notably, repeated Tdap immunizations in consecutive pregnancies have been well tolerated [80]. Following the recommendations for antenatal vaccination, an increasing trend of maternal vaccination coverage against pertussis has been reported. In 2020–2021, 53.5% of pregnant women were immunized with Tdap in the USA [81]. Uptake of Tdap vaccine depends on various factors, such as sociodemographic factors, access to healthcare providers, and maternal comorbidities [82]

Tdap immunization results in the production of mostly IgG1 immunoglobulin [83], which can be transferred easily through the placenta from the mother to the fetus; thus, neonates display higher B. pertussis-specific antibody titers than their mothers [84]. The effectiveness of maternal immunization against pertussis has been thoroughly described in the literature in high- and middle/low-income countries, reaching 91% [85,86,87,88,89,90].

The safety of pertussis vaccination during pregnancy has been well documented. Although pregnant women displayed an increased risk for fever and chorioamnionitis following pertussis vaccination, there was no association with an increased frequency of clinically related events for the baby or for the mother [91,92,93,94,95,96,97].

### 3.2. When to Vaccinate Mothers

Pregnant women are advised to receive the Tdap vaccine during the last trimester of gestation, due to the highest maternal IgG levels and the maximum transplacental transport of maternal immunoglobulins to the fetus after the 34th gestational week [61]. However, the optimal timing of maternal vaccination remains to be defined, while it seems that the sooner a pregnant woman gets vaccinated, the better the neonatal protection. The latter potentially implies that, in addition to a more robust maternal immunological response, the fetal immune system might have a longer period of time for stimulation [98,99,100,101,102]. Regarding antibody avidity, two studies reported higher avidity when pregnant women were immunized during the early third trimester than later [103,104], although this finding was not further validated [105].

### 3.3. In Utero Priming

Similarly to maternal influenza vaccination, the fetal immune response following tetanus and pertussis vaccination in pregnancy appears to expand beyond the transplacental maternal IgG transfer. In detail, studies have shown that, following maternal vaccination with tetanus toxoid vaccine, tetanus IgM was identified in their offspring, while IgM levels were correlated with the timing of maternal immunization, implying fetal B lymphocyte sensitization [106,107].

With regard to cellular immunity, data remain scarce. Lima et al. found that maternal Tdap immunization and higher pertussis-specific IgG antibody levels did not affect the cellular immune response to B. pertussis at birth, as no differences in the neonatal ex vivo challenge of B and T cells were observed [108]. However, this study did not investigate cellular responses at later timepoints after birth. Rice et al. demonstrated that Tdap vaccination induced distinct cytokine profiles in infants of immunized mothers against *B. pertussis* [109]. At birth, offspring of immunized women had elevated IL-2 and IL-12 whole-blood levels, increased classical monocyte frequencies, and reduced monocyte and NK cell cytokines. These findings suggest the possibility of in utero priming of the fetal immune system beyond IgG transplacental transfer. At 7 weeks of age, IL-2 remained elevated, while lower IL-10 and IL-13 responses were observed. Following primary DTaP series vaccination, babies of immunized women still displayed lower IL-10 and IL-4 responses. Persistently high levels of IL-2 could affect cellular immune response, while the reduction in Th2 cytokines in older infants may play a role in the attenuated humoral response following primary DTaP immunization [109]. Vaccine-induced maternal cytokines could be transferred via the placenta and directly modify the fetal and neonatal immune response [25,109,110].

### 3.4. Blunting Effect

Previous studies have shown that the immune response induced post primary series of pertussis vaccination among infants of vaccinated pregnant women is lower compared to infants of unvaccinated mothers and varies among different pertussis antibodies (PT, FHA, and PRN); however, this effect seems to diminish following the booster dose in the second year of age [13,111,112,113,114]. Remarkably, surveillance data from the United Kingdom did not report any resurgence of pertussis cases in the last months on infancy [115]. Therefore, this blunting effect may lack any clinical implications, although continuous monitoring is required.

## 4. Maternal Immunization against SARS-CoV-2

Since the emergence of severe acute respiratory syndrome coronavirus 2 (SARS-CoV-2) in December 2019 and the subsequent coronavirus disease (COVID-19), a new challenge has arisen for the public health globally, declared as the COVID-19 pandemic. Soon it became apparent that vaccination would be the most effective way to mitigate the burden of severe COVID-19. COVID-19 vaccines have received an emergency use authorization, including pregnant women, albeit initially excluded from clinical trials. Pregnant women present a higher risk of severe COVID-19 compared to their non-pregnant counterparts [62,116,117], mainly those with pre-existing conditions such as diabetes or hypertension, with higher rates of intensive care unit admission, invasive ventilation, and mortality [118,119]. The risk of perinatal complications such as preeclampsia, preterm labor, and fetus growth restriction is also increased [120]. Intrauterine transmission of the virus to the fetus is rare but postpartum neonatal infections are more prevalent, mainly presenting with respiratory distress, low oxygen saturation, and cough, with 38% of previously infected neonates requiring intensive care [121,122]. Thus, COVID-19 vaccination before or during pregnancy is crucial for the protection of both mothers and their offspring. Even during the era of the less severe Omicron variant disease, compared to previous variants, studies on the outcome of Omicron infection during pregnancy reported that moderate to severe disease can occur especially among those who are not vaccinated, highlighting the importance of vaccination, especially among the vulnerable pregnant women [123].

The Centers for Disease Control and Prevention and other professional organizations such as the ACOG and the Royal College of Obstetricians and Gynecologists (RCOG) highly recommend that pregnant and lactating women should receive immunization against COVID-19 when the benefits outweigh the potential risks [124,125,126,127]. According to the current recommendation, the available vaccines include Pfizer/BioNTech BNT162b2, Moderna mRNA-1273, AstraZeneca AZD1222, Johnson and Johnson/Janssen Ad26.COV2.S, Novavax NVX-Co2373, Sinopharm BIBP, Sinovac CoronaVac, and Bharat Biotech BBV152. In the USA, mRNA vaccines (Pfizer-BioNTech and Moderna) are preferred for primary vaccination and additional doses (for immunocompromised individuals), as well as booster vaccination. Thrombosis with thrombocytopenia syndrome (TTS) has been reported as an uncommon complication post immunization with the adenovirus-vectored vaccines AstraZeneca and Janssen. Nonetheless, there are currently no data supporting that pregnant women should be considered as a high-risk population for TTS compared to nonpregnant ones [128]. COVID-19 vaccines may be administered simultaneously with other vaccines such as influenza vaccine and Tdap which are routinely administered during pregnancy.

A meta-analysis for the uptake of COVID-19 vaccines in pregnant women among five countries covering the period from December 2020 to October 2021 reported that the overall percentage of vaccinated women did not exceed 27.5% [129]. Several factors, i.e., age, demographic and socioeconomic characteristics, and the fear for severe disease may contribute to vaccine hesitancy. Given the low prevalence of vaccine uptake against COVID-19 among pregnant women, it is imperative for governments to foster the trust in the vaccines and surpass the hurdle of vaccine hesitancy.

### Maternal Immunization Immunogenicity in Humans

Collier et al. were among the first to demonstrate that two doses of mRNA vaccine (BNT162b2 or mRNA-1273) received 3–4 weeks apart at various timepoints during pregnancy were immunogenic in pregnant women, while vaccine-elicited antibodies were detected in infant cord blood and mother’s breastmilk [130]. Another study showed that mRNA COVID-19 vaccines in pregnant women lead to antibody production in 5 days post first dose and transplacental transfer of IgG antibodies as early as 16 days post first dose [131]. Several subsequent studies confirmed the presence of maternal antibodies in cord blood, breast milk, and serum in infants of vaccinated mothers [132].

An anamnestic dose is now recommended for several high-risk populations, including pregnant women [133,134,135,136]. Individuals who received a third vaccine dose during the last trimester of pregnancy displayed higher levels of anti-spike IgG antibodies in maternal and cord blood [137]. This difference indicates that women with a primary series of vaccination followed by a booster dose transfer higher levels of antibodies to the neonate compared to those who received a two-dose schedule Interestingly, anti-spike IgG antibodies persist through the first 6 months of life, while IgG titers are higher at delivery, as well as 2 and 6 months postpartum, among infants born to mothers who were vaccinated at 20–32 weeks of gestation compared to those infants whose mothers were infected by SARS-CoV-2 during pregnancy at the same time interval [132]. More prospective and longitudinal studies are required in order to enlighten aspects such as the mechanisms of passive immunity after maternal vaccination, the exact duration of the provided immunity, and whether the antibodies passed by the mother have an adverse impact on the developing neonatal immune response [138].

Real-world data have confirmed vaccine effectiveness against hospitalization and admission to intensive care unit for COVID-19 among infants born to vaccinated mothers, most of them suggesting a stronger level of protection following the booster dose [139,140]. Interestingly, effectiveness was lower during the predominance of the omicron variant than during the delta circulation period as the omicron variant evades neutralizing antibodies induced by vaccination. Importantly, vaccine effectiveness against either variant was greater when the second dose of COVID-19 vaccine was administered after the 20th gestational week compared to earlier administration.

Further research is required to optimize maternal vaccination schedule, mainly regarding the booster dose, as well as define the level of subsequent protection in newborns. The main challenge remains to determine until when maternal vaccination may be postponed, considering the higher risk of severe COVID-19 during gestation.

## 5. Future Perspectives

Immunization against influenza, pertussis, and COVID-19 during pregnancy has been demonstrated to confer significant protection to both mothers [16,96,141] and young infants, and it should be offered to all pregnant women. Nonetheless, there are still issues to be addressed in this field. Immunization strategies should focus on maximizing the protective efficacy of maternal immunization while minimizing their inhibitory influence on infant B-cell responses. Further clinical trials are required to evaluate the long-term impact of maternal antibodies on memory B-cell induction and the potential skew of infant B-cell responses from immunodominant to non-immunodominant epitopes. Another promising hypothesis is the use of distinct vaccines in mothers and infants, for the recruitment of distinct B cells into the immunodominant response. Furthermore, it appears wise not to aim to higher maternal antibody titers than required for offspring protection. To this direction, the use of non-adjuvanted vaccines in pregnant women may be considered as an alternative [67]. Delaying immunization of infants of immunized mothers or offering additional/booster doses at an age at which maternal antibodies have declined below the threshold may be also considered.

Furthermore, in utero priming following maternal vaccination, if confirmed, could benefit the neonate, beyond protection through antibody-mediated passive immunity. This may be of great importance for cell-mediated infections, such as respiratory syncytial virus (RSV) [142]. RSV disease is responsible for severe respiratory distress among infants and young children, otherwise healthy, associated with high rates of hospital admissions and visits at the emergency departments [143]. Currently, there is only a monoclonal antibody, palivizumab, for the prevention of RSV disease among very preterm infants or those with specific comorbidities such as congenital heart disease, congenital respiratory abnormalities, or neuromuscular disease [144]. Maternal vaccination during pregnancy and infant vaccination with monoclonal antibodies may protect this vulnerable population against RSV disease [145]. Several RSV vaccines are currently under phase 3 clinical trials. A maternal vaccine formulation using the pre-fusion conformation of RSV protein (pre-F) seems to be the most promising one, eliciting high titers of RSV neutralizing antibodies, protecting the offspring from severe disease [146]. In a phase 2b trial, it was shown to be well tolerated for the mother and produced robust neutralizing antibody responses in pregnant women with adequate transplacental transfer [147]. Nevertheless, the clinical impact of the elevation of neutralizing antibody titers is under investigation. Additionally, the question of whether this vaccine may induce in utero priming of the fetus and affect the cellular immune response of the offspring is yet to be deciphered. Outside of RSV, other vaccines candidates are currently under investigation for the protection against group B streptococcus (GBS) and cytomegalovirus (CMV) infection, as phase 2 trial results and enrollment in phase 3 trial are soon expected [17].

Lastly, one should consider that protection of the mother and the offspring starts long before pregnancy, through maintaining high levels of active and herd immunity against significant vaccine-preventable infectious diseases. Pregnant women represent a vulnerable population to several infections, especially considering that many of the corresponding vaccines are contraindicated during pregnancy. A representative example of the significance of vaccination prior to gestation is the devastating outcome of congenital rubella syndrome, which causes serious birth abnormalities. This highlights the importance of active immunity before pregnancy and herd immunity. However, currently, the latter is undoubtedly threatened by a period of general vaccine hesitancy and major backsliding on childhood vaccinations due to the COVID-19 pandemic. Completed immunization with all the recommended vaccines is of paramount significance to sustain herd immunity. In the context of resurgence of previously eradicated diseases, such as measles and polio [148,149,150], healthcare providers should focus on ensuring high vaccination rates and offering any missed vaccinations in order to maintain antibody titers above herd immunity levels.

## 6. Conclusions

Maternal vaccination is a successful but still underutilized way for the prevention of infectious diseases among childbearing women and their offspring. The importance of informing pregnant women of the potential benefits of their timely vaccination for both themselves and their offspring cannot be emphasized enough.

Remarkably, increasing evidence supports the significance of maternal immunization not only through passive immunity, but also through in utero priming of the developing immune system. However, further in-depth research is required to shed light on the underlying mechanisms and possible clinical implications. Recent advances suggest that in utero fetal priming following maternal vaccination could benefit the newborn by offering protection in ways beyond antibody-mediated passive immunity. Nevertheless, several issues remain to be elucidated regarding maternal vaccination i.e., the possible impact on subsequent infant vaccinations, their potential “nonspecific” effects, and how the design and timing of immunization may dictate prenatal priming.

As data accumulate on how and via which mechanisms maternal vaccination affects fetal immune system, other possible methods for measuring antigen-specific T cells should be considered. This could aid in interpreting priming following vaccination, as it is debatable whether the observed cellular responses necessarily mirror in utero sensitization. Improving our understanding of the perinatal and neonatal immune systems is essential for improving infant survival rates and the optimization of vaccination in pregnancy and in early life, especially in developing countries, where the burden of infectious diseases is the highest.

## Data Availability

As this is a review article, no data were collected from patients.
